# Prevalence of Post Tonsillectomy Haemorrhage at a Tertiary Care Centre in Nepal

**DOI:** 10.31729/jnma.5831

**Published:** 2021-02-28

**Authors:** Prashant Tripathi, Rohita Bajracharya, Kunjan Acharya, Bijaya Kharel, Yogesh Neupane, Heempali Dutta, Kripa Dongol, Urmila Gurung

**Affiliations:** 1 Department of ENT and Head and Neck Surgery, Tribhuvan University Teaching Hospital, Institute of Medicine, Kathmandu, Nepal

**Keywords:** *adenoidectomy*, *haemorrhage*, *tonsillectomy*, *tonsillitis*

## Abstract

**Introduction:**

Tonsillectomy is one of the common ENT surgical procedures. Post-tonsillectomy haemorrhage remains a frequent complication which can be potentially life-threatening. The objective of the present study was to calculate the prevalence of haemorrhage following a tonsillectomy at a tertiary care centre.

**Methods:**

It was a descriptive cross-sectional study performed by medical chart review of the patients who underwent tonsillectomy from January 2018 to December 2019 at the department of ENT-Head and Neck Surgery of Tribhuvan University Teaching Hospital. Ethical approval was obtained from the institutional review committee (Ref:-282(6-11) E2 076/077). Convenient sampling method was used. All patients of any age who had tonsillectomy for recurrent tonsillitis or tonsillar hypertrophy with or without obstructive sleep apnoea and no missing information on chart review were included in the study. Data were entered in MS-Excel 2007 and analyzed in rate and percentage.

**Results:**

Ten (5.18%) out of a total of 193 patients who underwent tonsillectomy had a post-tonsillectomy haemorrhage. All 10 (100%) were adults patients, operated for recurrent tonsillitis, and used electrocautery. It was common in male patients 7 (70%). All of the haemorrhages was between a third and sixth postoperative day and were mild in severity.

**Conclusions:**

The prevalence of post-tonsillectomy haemorrhage was high at our centre during the study period of two years. It was common in adults, males and surgery done for recurrent tonsillitis using electrocauterization.

## INTRODUCTION

Tonsillectomy is a surgical procedure which involves removal of a tonsil or tonsils. It is one of the most commonly performed surgical procedures in otolaryngology practice.^1^ The purpose of surgery is to decrease antibiotic use, reduce leave from work or studies, hampering in daily activities, and, hence, an overall better quality of life.^2^

Major concerns after surgery are postoperative pain and post-tonsillectomy haemorrhage (PTH). Older age, chronic tonsillitis, excessive intraoperative blood loss and high postoperative mean arterial pressure are common risk factors for PTH.^3^ It is a significant consequence of tonsillectomy that can range from blood-tinged mucus to life-threatening bleeding.^4,5^

Clinical practice guidelines recommend performing an audit on PTH by all the institutions doing tonsillectomy to compare with national and international rates.^6^ The objective of the present study was to calculate the prevalence of haemorrhage following a tonsillectomy at a tertiary care centre in Nepal.

## METHODS

A descriptive cross-sectional study was carried out by a retrospective review of the medical records of patients who underwent tonsillectomy with or without adenoidectomy at the ENT-Head and Neck Surgery Department of Tribhuvan University Teaching Hospital (TUTH) from January 2018 to December 2019. Ethical approval was obtained from the institutional review committee of the Institute of Medicine (IOM), Nepal (Ref:-282(6-11) E2 076/077). The sample included adults and children of both genders that were operated on during the study period for recurrent tonsillitis or tonsillar hypertrophy with or without obstructive sleep apnoea syndrome. Patients with a bleeding disorder, those taking medication that may interfere with bleeding like Aspirin, Clopidogrel and those undergoing tonsillectomy as a part of uvulopalatopharyngoplasty for snoring were excluded from the study. Similarly, those with incomplete data or no medical record available were also excluded from the study.

The sample size was calculated based on the article published from study done at Dhulikhel hospital in which the prevalence of PTH was 12.6%.^9^ Using the formula for sample size calculation


$$\begin{array}{ll} \text{n} & ={\text{Z}}^{\text{2}}\times \text{p}\times \text{q}/{\text{e}}^{\text{2}}\\ & ={\left(1.96\right)}^{2}\times (0.126\times .874)/{\left(0.05\right)}^{\text{2}}\\ & =170 \end{array}$$

where,

Z = 1.96 at 95% Confidence Interval
p = prevalence, 0.1269
q = 1-p
e = margin of error, 5%

Hence, the required sample size was 170, but we included all 193 patients whose medical record was available for review.

In this study, PTH was defined as any bleeding after tonsillectomy procedure. In cases of adenotonsillectomy, PTH was considered where the bleeding was seen to come from tonsil bed. The PTH was considered primary if bleeding occurred within 24 hours of surgery and secondary if it occurred after 24 hours postoperatively.^7^ Severity of PTH was graded as follows: Grade I (Minor) -Haemorrhage that could be controlled by non-invasive intervention; Grade II (Moderate)-Haemorrhage necessitating local anaesthesia for control and Grade III (Severe)-Administration of general anaesthesia is necessary to control haemorrhage.^4^ All those patients who bled during the hospital stay period after the operation or who were readmitted because of PTH were included in the study.

Performa was prepared that included the recording of patient's particulars, surgical indication for tonsillectomy, type of surgery, the experience of the surgeon (resident or faculty), time of haemorrhage and control method administered for bleeding. Data were entered in MS-EXCEL and analyzed in rate and percentage whenever required.

## RESULTS

From January 2018 to December 2019, a total of 193 tonsillectomies performed at ENT-Head and Neck Surgery Department of TUTH met the inclusion criteria to be included in the study. Out of those operated cases, 10 (5.18%) patients had post-tonsillectomy haemorrhage (PTH). The bleeding occurred from third to fifth postoperative day. During this period, the post-tonsillectomy bleeding was of mild (Grade I) in severity with minimal oozing from the tonsillar fossa controlled by removal of clots and packing of tonsillar fossa with adrenaline-soaked gauze followed by hydrogen peroxide gargle. Neither of these patients required to be taken to operation theatre nor required blood transfusion. Tranexamic acid was also given parenterally in some cases. Out of ten patients who bled one was hypertensive, and others had no other co-morbid condition.

There was a total of 108 children and 85 adults. The average age for the adult who underwent tonsillectomy was 27.9 years (15-54 years), and for children, it was 6.1years (2-15 years). The youngest operated being two years and the eldest being 54 years. None of the patients had bleeding following tonsillectomy in the pediatric age group while in the adult age group 10 (11.8%) of the total operated patients had following bleeding tonsillectomy (Table 1).

**Table 1:** Post tonsillectomy haemorrhage in paediatric and adult age groups.

Age	Number (n=193)	PTH (n=10) n (%)
Children (≤15years)	108	0 (0)
Adults (≥15 years)	85	10 (11.8%)

**Figure 1.**
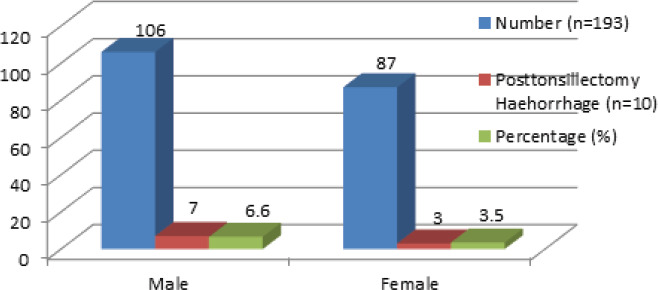
Post-tonsillectomy haemorrhage prevalence in male and female.

There were 106 males and 87 females operated; out of which 7 (70%) males and 3 (30%) females had post-tonsillectomy bleeding (Figure 1). The rate of PTH among males was 6.6% while it was 3.5% among females.

Out of 193 tonsillectomies, 88 (45.60%) tonsillectomies were performed for recurrent tonsillitis and 105 (54.40%) for tonsillar hypertrophy with or without obstructive sleep apnea syndrome (OSAS). Majority of adult patients had tonsillectomy for recurrent tonsillitis while a majority of children had it for tonsillar hypertrophy leading to OSAS. None of the patient that underwent tonsillectomy for tonsillar hypertrophy experienced PTH. However, 10 (11.4%) of the patient that underwent an operation for recurrent tonsillectomy had postoperative bleeding (Table 2).

**Table 2:** Post tonsillectomy haemorrhage in patients with different indications of tonsillectomy.

Indication of tonsillectomy	Number (n=193)	PTH (n=10) n (%)
Recurrent Tonsillitis	88	10 (11.4)
Tonsillar Hypertrophy	105	0 (0)

**Figure 2.**
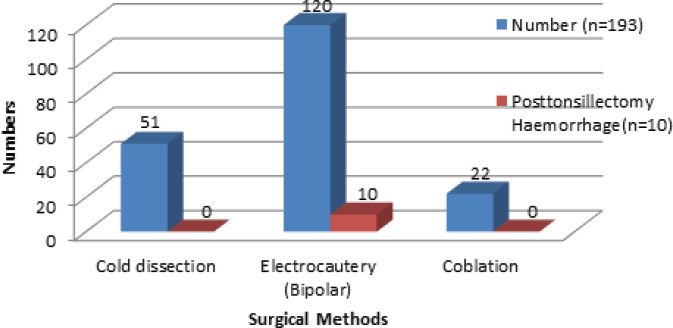
Post-tonsillectomy haemorrhage according to the surgical methods used for tonsillectomy.

Surgical methods used were cold dissection for 51 (26.43%) patients, electrocautery for 120 (62.18%) patients and coblation for 22 (11.40%) patients. All the post-tonsillectomy bleeding was found to occur in those patients that were operated on using bipolar electrocautery. It was found that 8.3% of total patients operated on by bipolar electrocautery experienced post-tonsillectomy bleeding (Figure 2).

Faculties had operated on 138 patients and residents operated on 55 patients. PTH occurred on 1.45% and 14.54% of those operated by faculties and residents, respectively (Table 3).

**Table 3:** Post tonsillectomy haemorrhage depending on the experiences of the surgeon.

Surgeon	Frequency	PTH n (%)
Faculties	138	2 (1.45)
Residents	55	8 (14.54)

## DISCUSSION

Post-tonsillectomy haemorrhage (PTH) has been considered one of the potentially life-threatening complications an ENT surgeon might face during surgical practice.^8^ Tonsillectomy is one of the initial procedures learnt by the beginners, and although viewed as a relatively low-risk procedure, it can be potentially harmful because of the chance of post-tonsillectomy haemorrhage.^8^ In a study done at Dhulikhel Hospital of Nepal, PTH was seen in 12.6% with one out of 15 PTH returning to the operation theatre.^9^ In the studies conducted in different centres and countries, the PTH varies from 3.3% to 13.9%.^10-12^ In a study conducted in Australia, PTH was observed in 6.9% of patients with 2.8% returning to the theatre.^10^ A review of 63 studies by Blakley et al. in 2009 came to the notion that the maximum acceptable PTH rate is 13.9%.^11^ In our study, the prevalence of PTH was 5.12% which was similar to other studies. All PTH were minor bleeding managed by conservative means. None of the patients having PTH during study period required to be returned to the operation theatre.

Studies done to identify causes for PTH have largely variable results. We also did various subgroup calculation of PTH prevalence like age group, gender, indication for surgery, the method used for tonsillectomy, and the operating surgeon's experience. A study has suggested that postoperative clot sloughing or infection as a potential cause of secondary PTH.^10^ Several studies show conflicting results in the role of gender as a risk for PTH. Some studies show male gender having a significantly greater risk for PTH.^13,14^ In our study, as in other studies, the frequency of PTH was 6.6% among the male patients while it was 3.5% among the female patients. There are also varying reports regarding the influence of age on PTH.^3,4^ Generally, the frequency of PTH is higher in older children or adults as seen in our study.^3,10^ This difference could be due to difference in the indication for the tonsillectomy. In adults, tonsillectomy is done more frequently for recurrent tonsillitis which has scarring and leads to increased trauma during surgery which can cause PTH. Similarly, in our department, the paediatric tonsillectomy is done by faculties which are more experienced than the in-the-training resident doctors and hence reduced rate of complications.

Patients undergoing tonsillectomy for recurrent tonsillitis seem to have a higher likelihood of PTH than other indications for tonsillectomy such as obstructive sleep apnoea.^3,5,15,16^ In our study also we noted PTH exclusively in patients who underwent surgery for recurrent tonsillitis. This is possibly associated with greater intra-operative trauma due to scarring from recurrent tonsillitis resulting in subsequent clot sloughing postoperative period.^16^

The role of surgical technique is a continuous debate as a risk factor for post-surgery bleeding. However; we found all bleeding occurred in patients who underwent tonsillectomy by bipolar electrocautery diathermy. It may be because of excessive cautery used for haemostasis leading to a higher likelihood of clot sloughing. The infection follows the thermal damage with the use of electrosurgical techniques by the enzyme and bacteria containing saliva, leading to PTH.^17^ The use of electrosurgery was associated with a higher incidence of PTH as reported by different studies.^1,12,18^ Coblation technique for tonsillectomy is currently used by a more experienced surgeon and more often in paediatric tonsillectomy, which may be the reason for lack of PTH in this group.

Some studies showed an association between surgical experience and PTH rates.^19^ However, several others have shown no statistically significant differences with regard to the surgeon's experience.^3,8,10^ In this study, out of 55 patients operated by residents, 14.54% had PTH while out of 138 patients operated by faculties, only 1.45% had PTH. This may be because, with the experience, the operative time and the unintentional trauma to the tonsil bed and pillar are reduced.

The drawback of this type of retrospective study is that it depends upon the accuracy of records. Moreover, the rate of PTH may be under-reported as many patients who experience scanty bleeding after discharge may not come to the hospital or get readmitted. The other limitations of this study are a small number, hospital-based data with no comparison or control group. This can carry the risk of bias as there could be missing of the recording as well as underreporting. But, still, this can provide insight into further study.

## CONCLUSIONS

The prevalence of post-tonsillectomy bleeding in our study was high. The bleeding was more commonly seen in tonsillectomy done in adult and male patients with electrocautery and for recurrent tonsillitis. Similarly, bleeding was more common in tonsillectomy done by residents. Studies are done prospectively and with a larger sample and at multiple centres can provide more accurate data.
